# Supplementary material for: The fecal resistome of dairy cattle is associated with diet during nursing
Source: Nat Commun. 2019 Sep 27;10:4406. doi: 10.1038/s41467-019-12111-x (PMC6765000; doi:10.1038/s41467-019-12111-x)
Supplement: Supplementary file 1 — Supplementary Information [file 41467_2019_12111_MOESM1_ESM.pdf]

The fecal resistome of dairy cattle is associated with diet during nursing

Liu et al.

Supplementary Information

Supplementary Figures

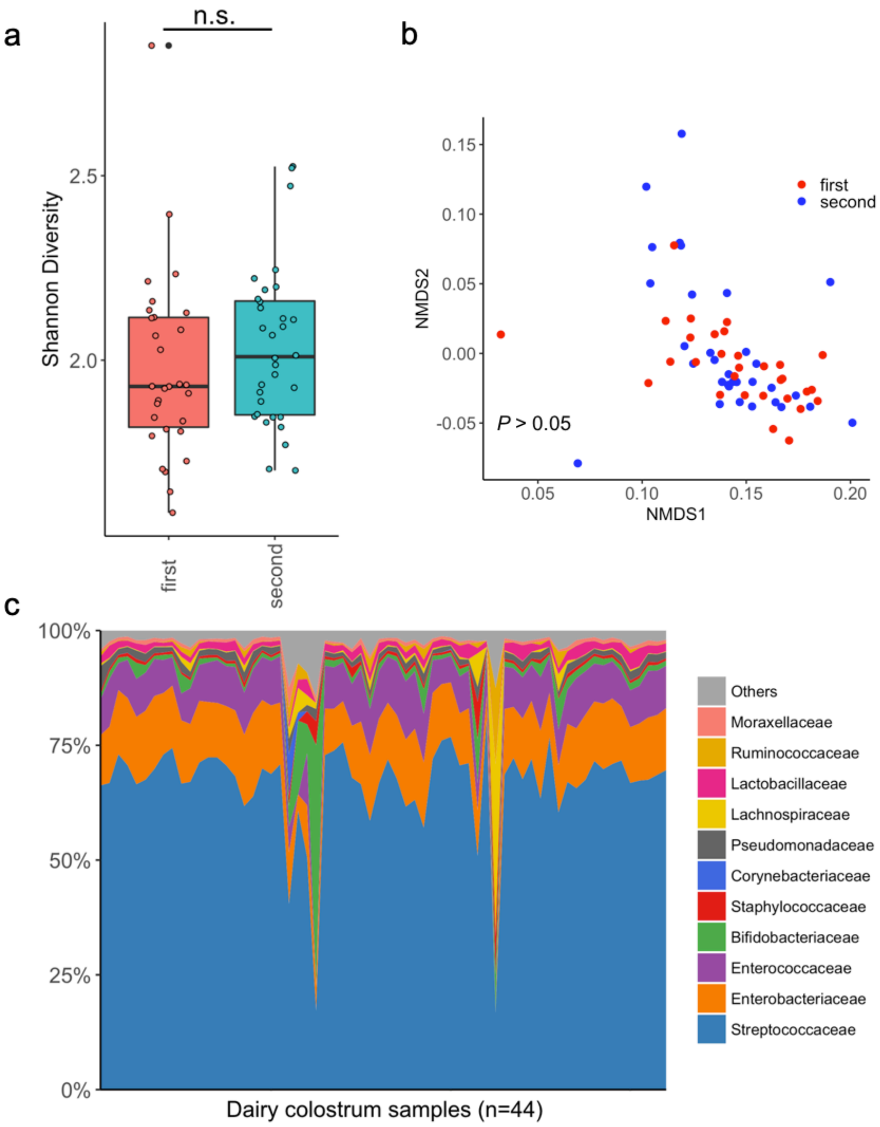

Supplementary Figure 1: The first and second colostrum fed to dairy calves exhibited similar microbiota. (a) Boxplot of Shannon diversity of colostrum. The Shannon index values were presented as the median (central black horizontal line); the lower and upper hinges correspond to the 25th and 75th percentiles. (b) NMDS analysis (dimension = 3, stress = 0.04). (c) Relative abundance of bacterial family detected in dairy colostrum. n.s. indicates $P > 0.05$ by a Kruskal-Wallis test.

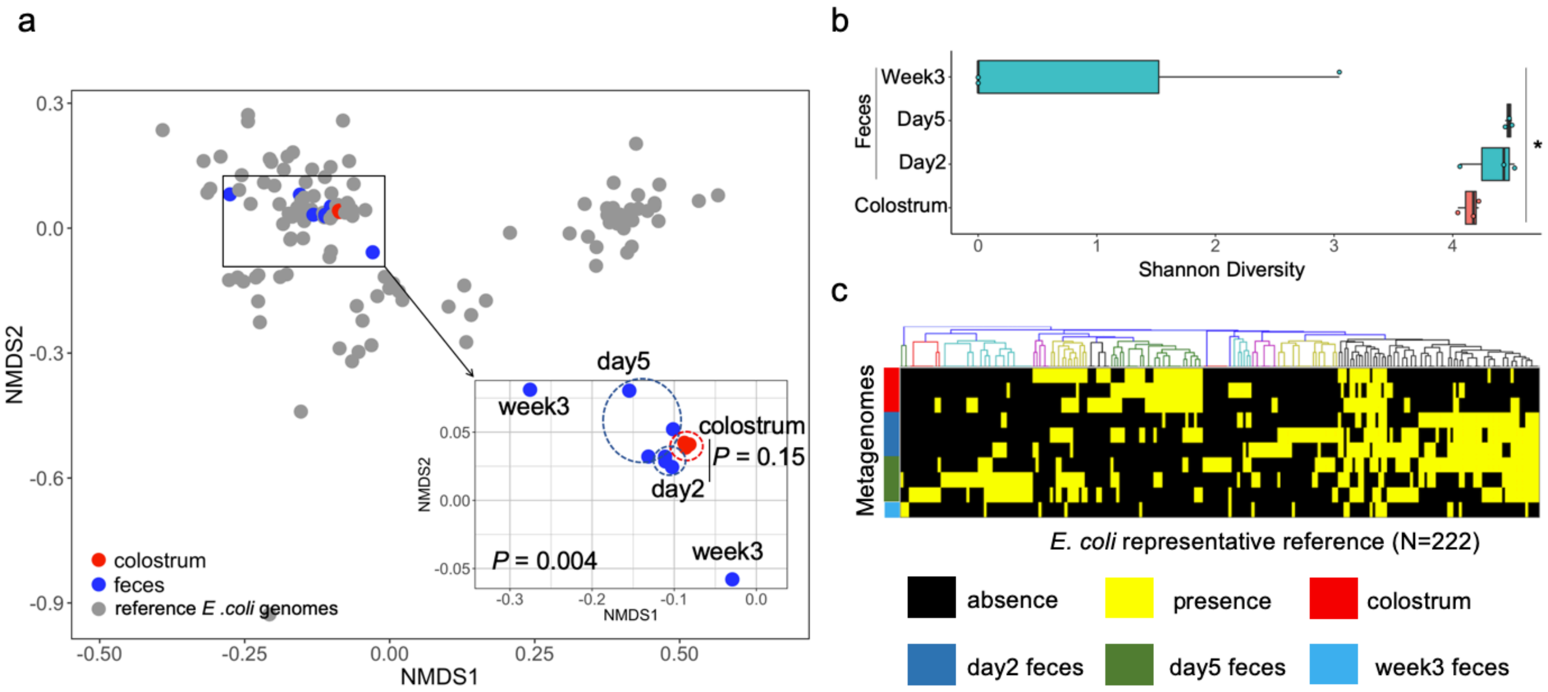

Supplementary Figure 2: Metagenomic strain profiling in colostrum and dairy calf fecal samples. (a) 3 dimensional NMDS of the genetic profile of the 118 reference strains (PanPhlAn) and the 11 dominant *E. coli* strains (this study) based on a Jaccard dissimilarity calculation (stress = 0.1). Colostrum samples (n=3) were circled in red, day 2 (n=3) and day 5 (n=3) fecal samples were circled in blue. *E. coli* strains were below our detection limit in one week 3 sample and all week 7 fecal samples (n=3) with the PanPhlAn analysis. Pairwise permutation MANOVAs (post-hoc test for multiple comparisons) were performed in R with the RVAideMemire package, and significant differences between groups were evaluated after FDR adjustment for α = 0.05. (b) Boxplots (boxes representing IQRs with median shown in black) showing the calculated Shannon diversity of coexisting *E. coli* strains (StrainEst) identified in colostrum and fecal metagenomes. *E. coli* strains were below our detection limit in two week 3 samples and all week 7 fecal samples (n=3) with the StrainEst analysis. *$P < 0.05$ by Friedman's test. (c) Heatmap depicting the presence or absence of specific *E. coli* strains in metagenomes. There are 222 *E. coli* representative references (out of 278 characterized representative *E. coli* in StrainEst) were identified in our study. Hierachical clustering was accomplished using script hclust2.py at [https://bitbucket.org/nsegata/hclust2]. The sequencing reads from the first and second colostrum samples were analyzed together for both metagenome strain profiling analyses. Source data are provided as a Source Data file.

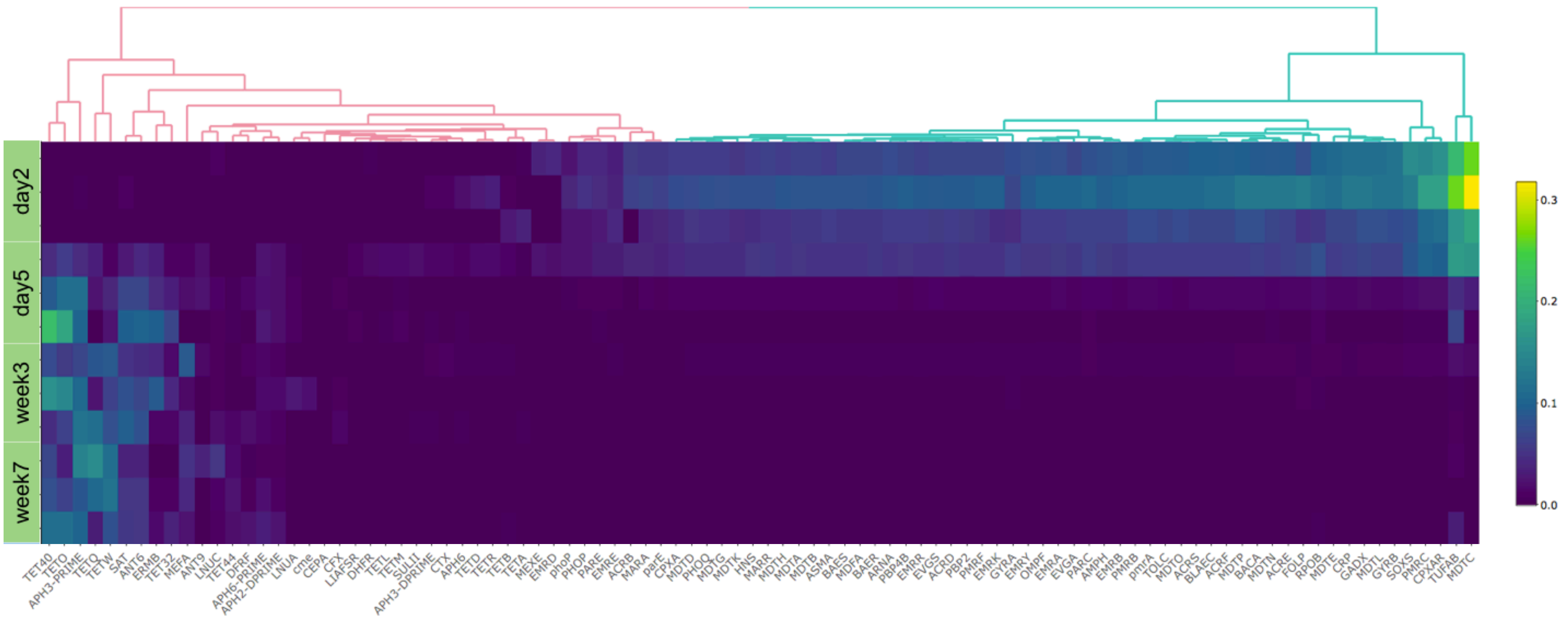

Supplementary Figure 3: Heatmap of normalized abundance of ARG groups in dairy calves. Groups of ARGs (n=45; represent 32.4% total observed ARGs groups) with an averaged abundance less than 9 X 10^{-4} were omitted from this plot for better visualization. Detailed information of these ARGs is listed in Supplementary Data 1.

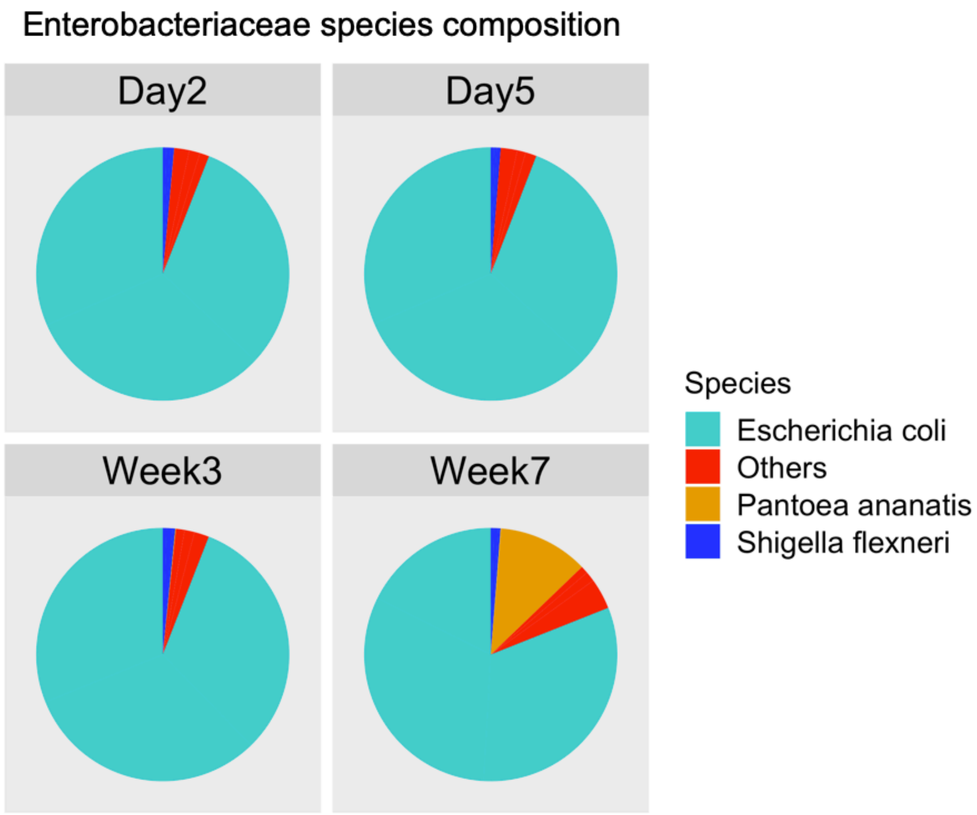

Supplementary Figure 4: Distribution of species within the family Enterobacteriaceae over time. The taxonomy classification was performed using Kraken2 to against a custom database including RefSeq and 4941 metagenome-assembled rumen genomes. The relative abundance of species within Enterobacteriaceae was estimated using Bracken.

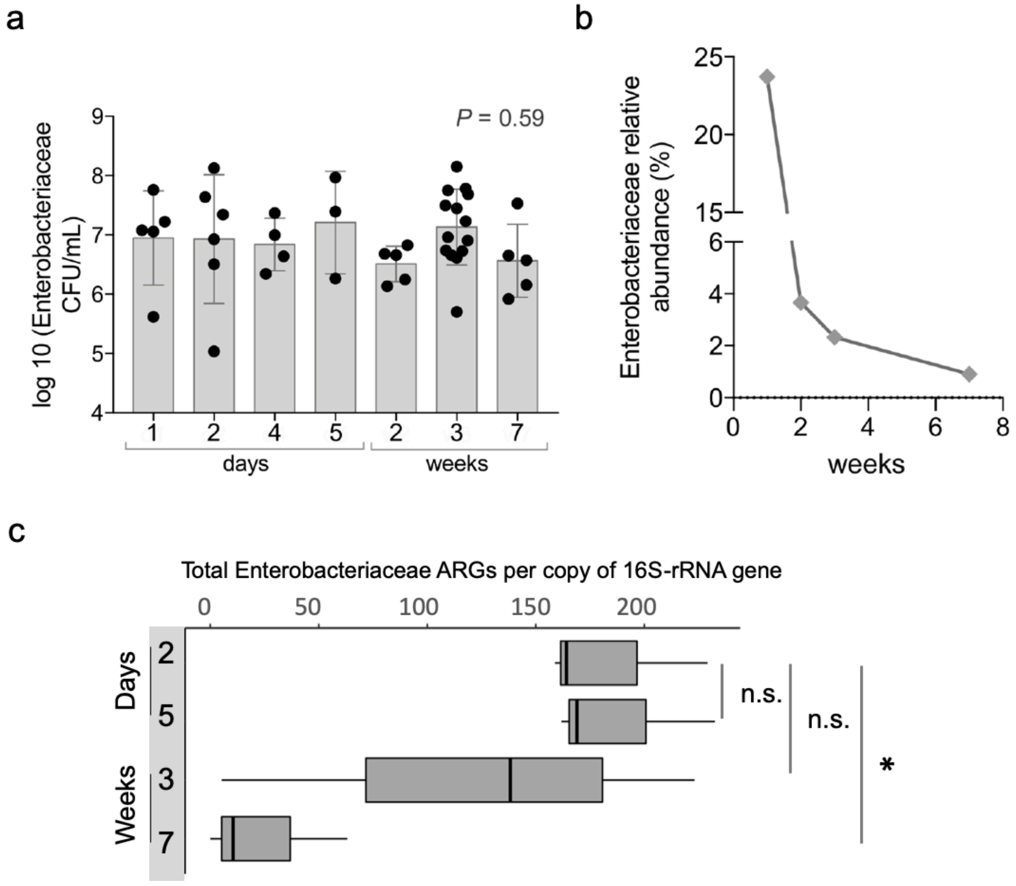

Supplementary Figure 5: Quantification of Enterobacteriaceae and its antibiotic resistance in dairy calves. (a) Bar chart showing the absolute abundance of Enterobacteriaceae over time based on qPCR. Error bar represents the standard deviation. (b) Line chart showing Enterobacteriaceae relative abundance from the16S rRNA gene sequencing data. (c) Boxplots (boxes representing IQRs with median shown in black) showing the total abundance of ARGs predicted to originate in Enterobacteriaceae per copy of 16S-rRNA gene over time. This was calculated by using metagenomic sequencing reads aligned to Enterobacteriaceae to run through AMR++ pipeline. *P < 0.05, and n.s. indicates P > 0.05 by Friedman's test followed by multiple pairwise comparisons using Nemenyi post-hoc test.

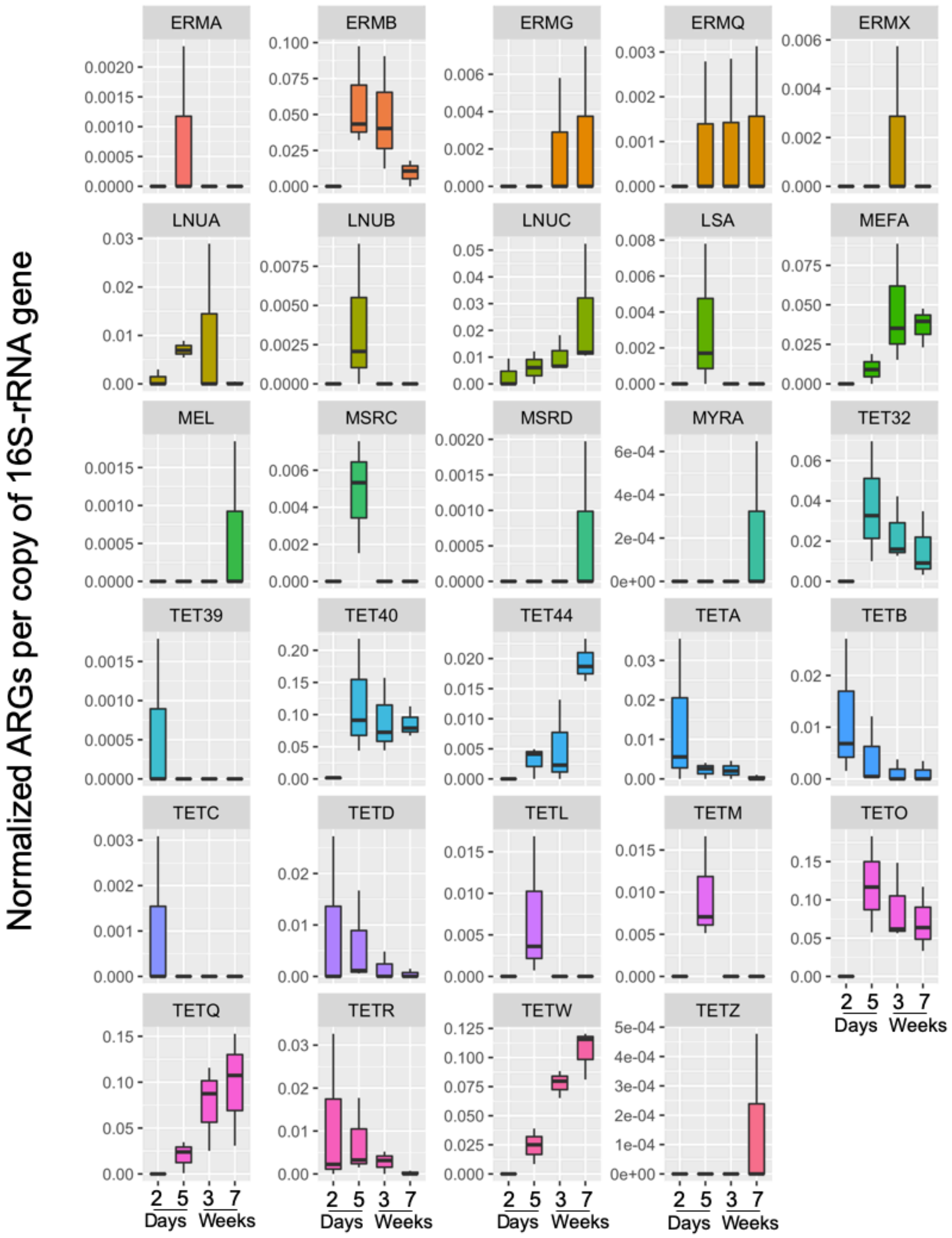

Supplementary Figure 6: Gradually increased ARGs to MLS and tetracycline in dairy calves. Boxplots (boxes representing IQRs with median shown in black) depicting the time-series abundance distribution of individual ARG group conferring resistance to MLS and tetracycline. Analysis was based on the normalized ARGs counts from the AMR++ pipeline. Descriptive information concerning ARGs is listed in Supplementary Data 1.

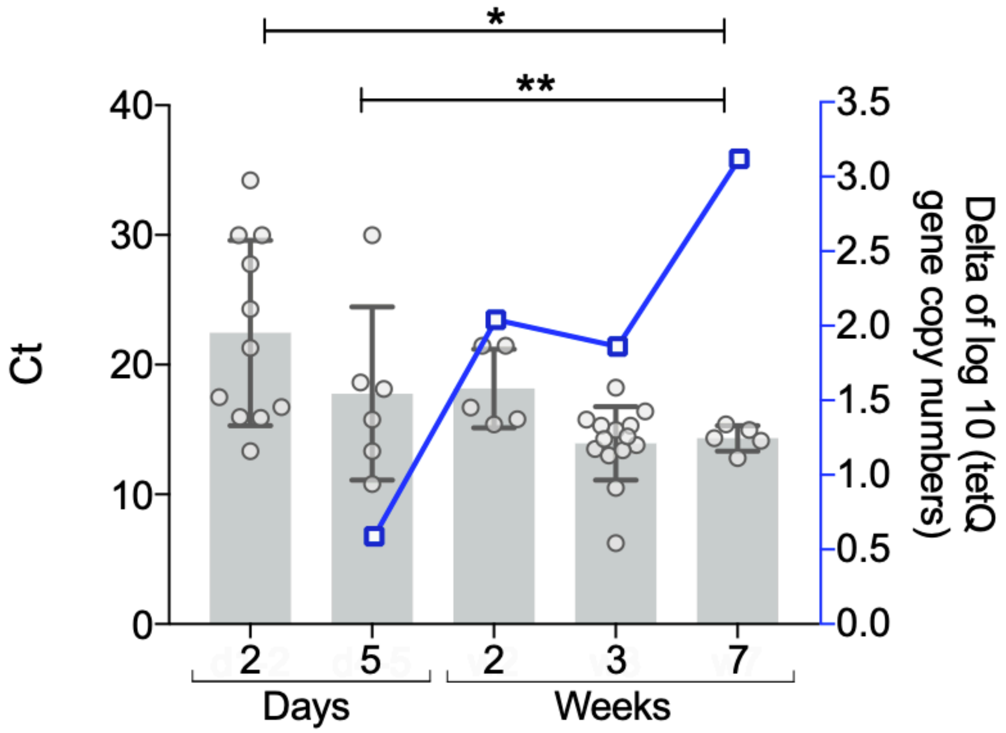

Supplementary Figure 7: The absolute quantification of *tetQ*. Ct values were directly obtained from the qPCR assay, and delta-Ct values were calculated to monitor the dynamic change of the abundance of *tetQ*. Error bar represents the standard deviation. One-way ANOVA was used to assess the Ct values over time. Significant differences were observed between the samples collected at days 2 and 5 and the samples collected at week 7, *$P < 0.05$ and **$P < 0.01$.

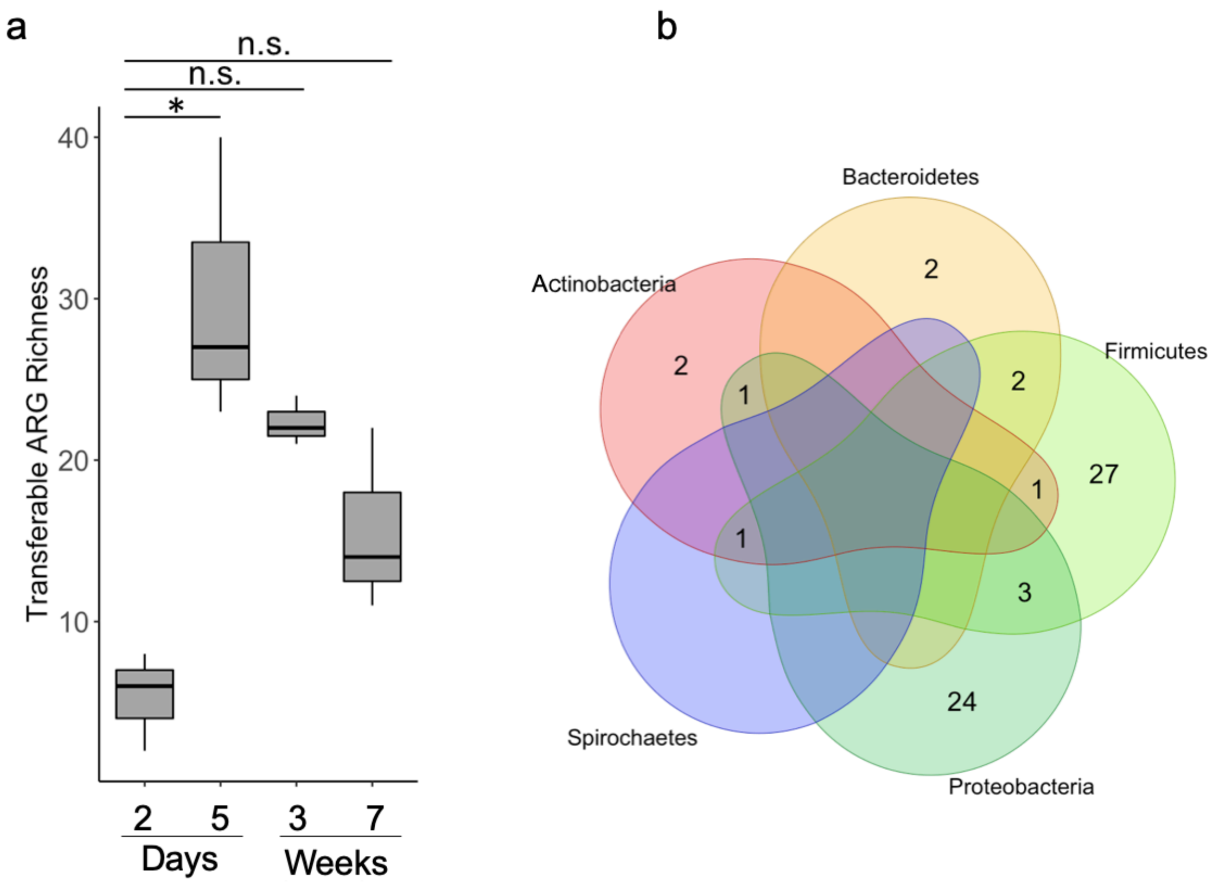

Supplementary Figure 8: The distribution of observed transferrable ARGs across bacterial phyla in dairy calves. (a) Boxplot (boxes representing IQRs with median shown in black) of the richness of transferrable ARGs in dairy calves over time. (b) Venn diagram depicting shared ARGs across taxa; colors were used to distinguish different phyla. Data were obtained from the ResFinder analysis (see Methods). **P* < 0.05, and n.s. indicates *P* > 0.05 by Friedman's test followed by multiple pairwise comparisons using Nemenyi post-hoc test.

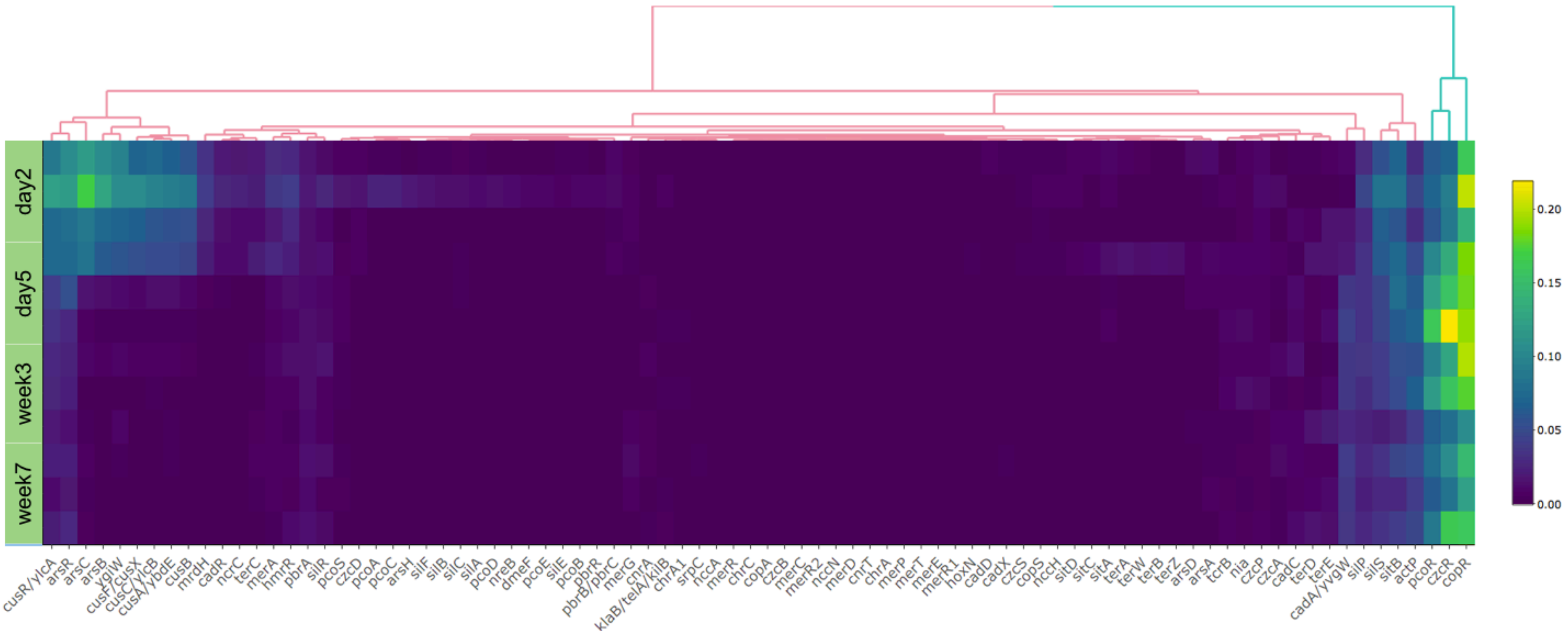

Supplementary Figure 9: Heatmap of normalized abundance of heavy metal resistance genes in dairy calves. MRGs (n=20; represent 19.2% total observed MRGs) with an averaged abundance less than 8.62 X 10^{-5} were omitted from this plot for better visualization. Descriptive information of MRGs is listed in Supplementary Data 2.

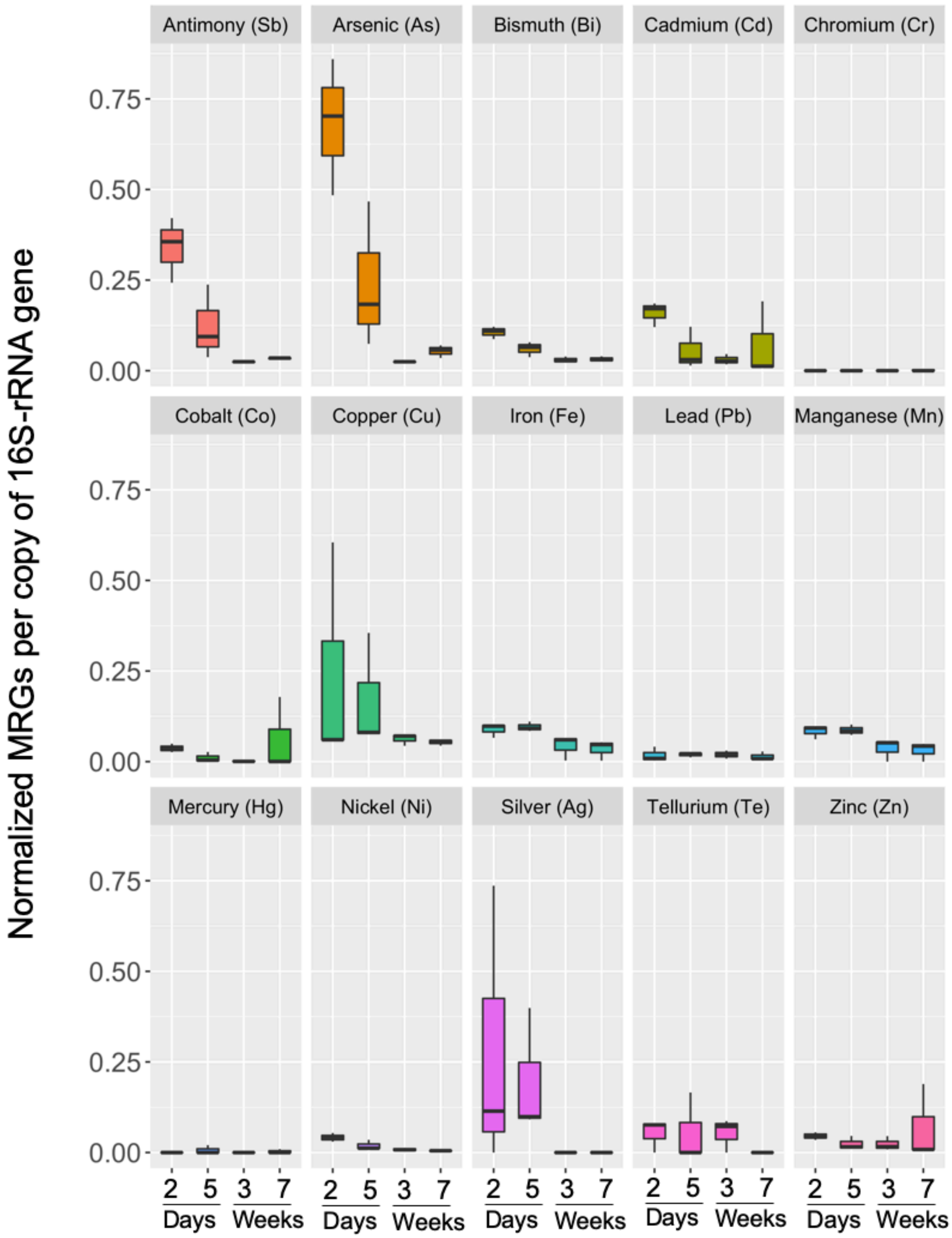

Supplementary Figure 10: The distribution of MRGs in dairy calves. Boxplot (boxes representing IQRs with median shown in black) of the distribution of normalized abundance of metal resistance genes (MRGs) conferring resistance to 15 metal compounds over time in dairy calves.

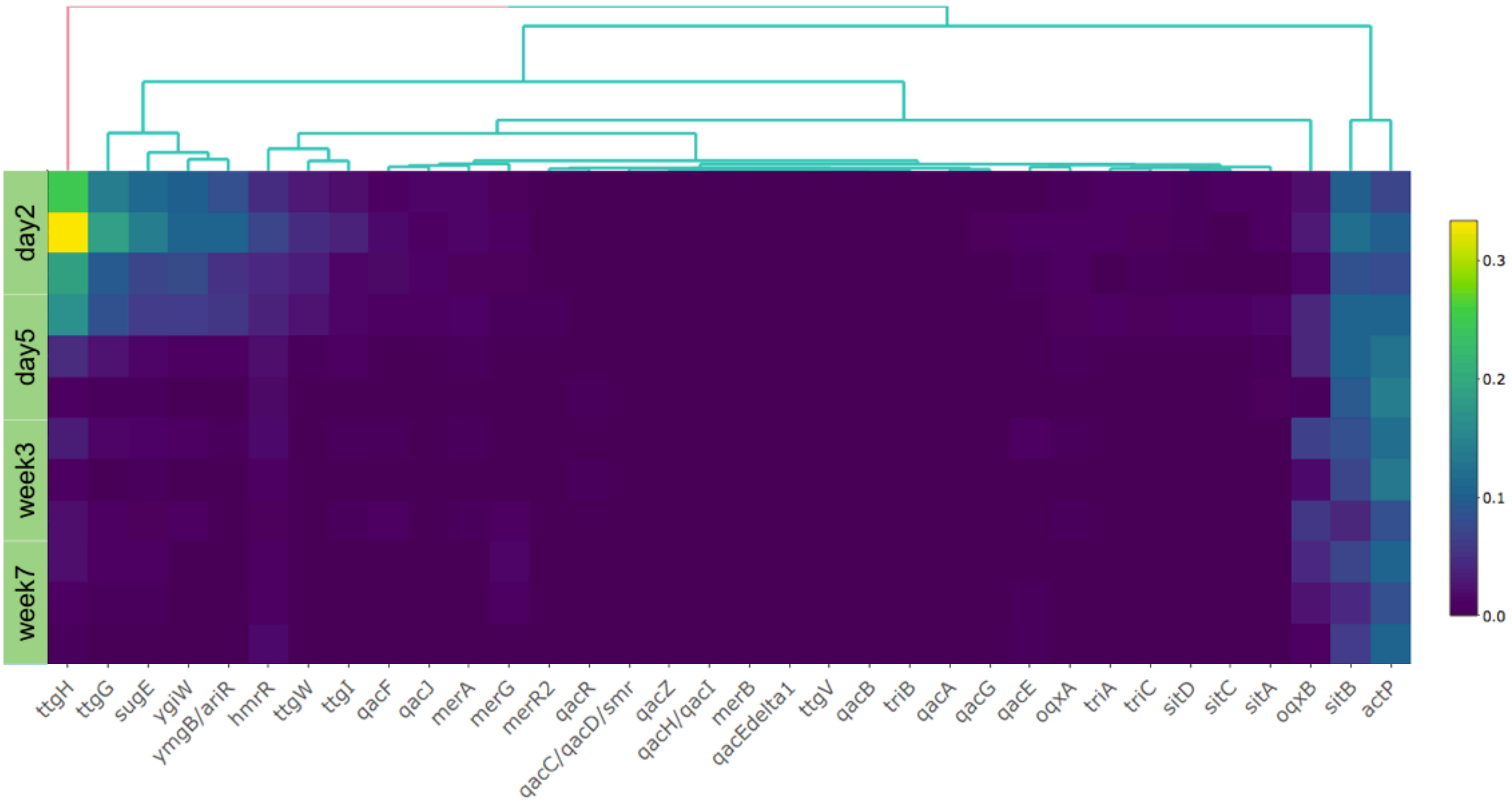

Supplementary Figure 11: Heatmap of normalized abundance of biocide resistance genes in dairy calves. Descriptive information of BRGs is listed in Supplementary Data 2.

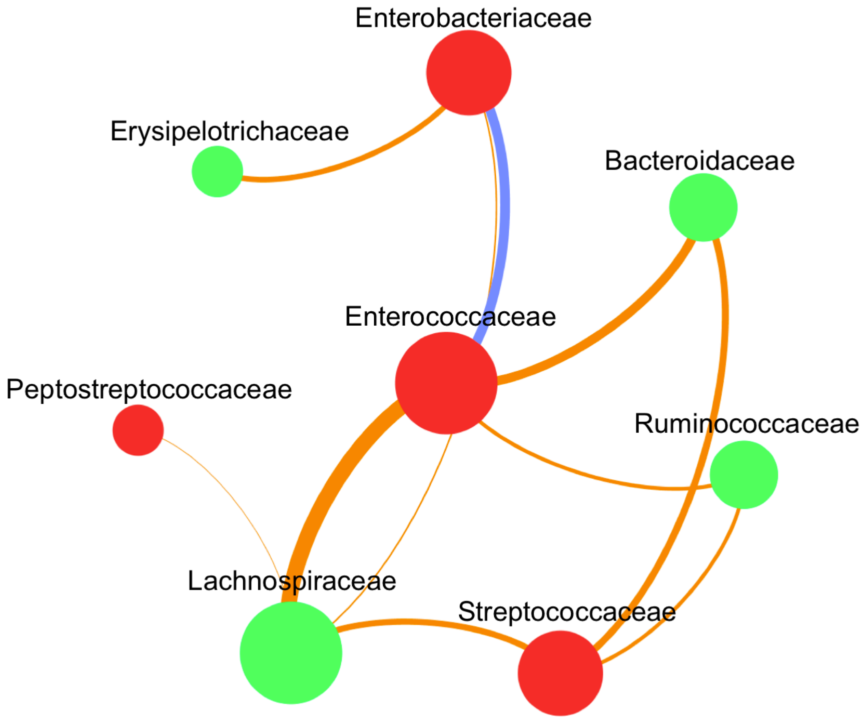

Supplementary Figure 12: Dairy calf fecal microbe-microbe interaction network at the family level. Nodes are microbes which were classified to the family level, and the size represents the number of connections (degree). Nodes colored red are the organisms which were predicted to harbor more ARGs, and green nodes are bacteria which are equipped with fewer ARGs. An orange edge represents a negative correlation while blue edge is a position correlation.

Supplementary Tables

Bacterial Group / Gene	Primer name	Sequence (5'-3')	Tm(°C)	Reference
Enterobacteriaceae	Entero-F<br>Entero-R	CATTGACGTTACCCGCAGAAGAAGC<br>CTCTACGAGACTCAAGCTTGC	56	(Oh *et al.*, 2012)
tetQ	tetQ-F<br>tetQ-R	AGAATCTGCTGTTTGCCAGTG<br>CGGAGTGTCAATGATATTGCA	63	(Aminov *et al.*, 2001)

Supplementary Table 1: List of qPCR primers and annealing temperatures (Tm) used in this study.
