# Supplementary material for: The fecal resistome of dairy cattle is associated with diet during nursing
Source: Nat Commun. 2019 Sep 27;10:4406. doi: 10.1038/s41467-019-12111-x (PMC6765000; doi:10.1038/s41467-019-12111-x)
Supplement: Supplementary file 3 — Description of Additional Supplementary Files [file 41467_2019_12111_MOESM3_ESM.pdf]

Description of Additional Supplementary Files

File Name: Supplementary Data 1
Description: List of ARGs observed in this study.

File Name: Supplementary Data 2
Description: List of BMRGs observed in this study.
