# Supplementary material for: The fecal resistome of dairy cattle is associated with diet during nursing
Source: Nat Commun. 2019 Sep 27;10:4406. doi: 10.1038/s41467-019-12111-x (PMC6765000; doi:10.1038/s41467-019-12111-x)
Supplement: Supplementary file 6 — Reporting Summary [file 41467_2019_12111_MOESM6_ESM.pdf]

nature research

Corresponding author(s): David Mills (University of California, Davis)

Reporting Summary

Nature Research wishes to improve the reproducibility of the work that we publish. This form provides structure for consistency and transparency in reporting. For further information on Nature Research policies, see Authors & Referees and the Editorial Policy Checklist.

Statistical parameters

When statistical analyses are reported, confirm that the following items are present in the relevant location (e.g. figure legend, table legend, main text, or Methods section).

n/a	Confirmed	
☐	☒	The exact sample size (*n*) for each experimental group/condition, given as a discrete number and unit of measurement
☐	☒	An indication of whether measurements were taken from distinct samples or whether the same sample was measured repeatedly
☐	☒	The statistical test(s) used AND whether they are one- or two-sided<br>*Only common tests should be described solely by name; describe more complex techniques in the Methods section.*
☐	☒	A description of all covariates tested
☐	☒	A description of any assumptions or corrections, such as tests of normality and adjustment for multiple comparisons
☐	☒	A full description of the statistics including central tendency (e.g. means) or other basic estimates (e.g. regression coefficient) AND variation (e.g. standard deviation) or associated estimates of uncertainty (e.g. confidence intervals)
☐	☒	For null hypothesis testing, the test statistic (e.g. *F*, *t*, *r*) with confidence intervals, effect sizes, degrees of freedom and *P* value noted<br>*Give P values as exact values whenever suitable.*
☒	☐	For Bayesian analysis, information on the choice of priors and Markov chain Monte Carlo settings
☐	☒	For hierarchical and complex designs, identification of the appropriate level for tests and full reporting of outcomes
☒	☐	Estimates of effect sizes (e.g. Cohen's *d*, Pearson's *r*), indicating how they were calculated
☐	☒	Clearly defined error bars<br>*State explicitly what error bars represent (e.g. SD, SE, CI)*

Our web collection on statistics for biologists may be useful.

Software and code

Policy information about availability of computer code

Data collection	No software was used for data collection.
Data analysis	PEAR (0.9.8), FASTX (0.0.14), cutadapt (1.8.3), QIIME (1.9.1), R (3.4.1), Gephi (0.9.2), bmtools (version 1), FLASH (1.2.11), Trimmomatic (0.36), MEGAHIT (1.0.6), MEGARes (1.0.1), ResFinder (2.1), BacMet (1.1), ResistomeAnalyzer (version 1), METAXA2 (2.1.3), DeSeq2 (3.8), RefSeq, CAZy, DIAMOND (0.9.22), PanPhlAn, StrainEst (1.2.4), taxator-tk (1.3.3), Kraken2, Bracken.

For manuscripts utilizing custom algorithms or software that are central to the research but not yet described in published literature, software must be made available to editors/reviewers upon request. We strongly encourage code deposition in a community repository (e.g. GitHub). See the Nature Research guidelines for submitting code & software for further information.

Data

Policy information about availability of data

All manuscripts must include a data availability statement. This statement should provide the following information, where applicable:

- Accession codes, unique identifiers, or web links for publicly available datasets
- A list of figures that have associated raw data
- A description of any restrictions on data availability

Sequencing data generated from both amplicon and shotgun metagenomes in this study have been deposited with the NCBI SRA (PRJNA438833) and are publicly available.

Field-specific reporting

Please select the best fit for your research. If you are not sure, read the appropriate sections before making your selection.

☒ Life sciences ☐ Behavioural & social sciences ☐ Ecological, evolutionary & environmental sciences

For a reference copy of the document with all sections, see nature.com/authors/policies/ReportingSummary-flat.pdf

Life sciences study design

All studies must disclose on these points even when the disclosure is negative.

Sample size	A total of 22 newborn dairy calves were enrolled to profile the gut microbiota during the first 10 weeks. The subject numbers were determined based on published studies/literature review, animals from the same farm of same age have very similar gut microbiota, a number of 22 is sufficient for us to handle subject-subject variances. In addition, samples from a total of 3 calves were included in a longitudinal metagenomic analysis.
Data exclusions	All collected rectal swabs and colostrum samples were included in the 16S rRNA gene sequencing portion of the study. A subset was randomly chosen for the metagenomics section.
Replication	For 16S analysis, we have individual samples collected from 22 subjects which serve as the biological replicates to each other. For shotgun metagenomics, we have three biological replicates per time with limited variances and we are confident of the reproductivity.
Randomization	From Apr. to Sep.of 2015, all healthy new born calves from dams in the UC Davis herd were included in this study. Three calves were chosen randomly from all included calves for the metagenomic analysis. Given this was a longitudinal study, calves were necessarily re-sampled.
Blinding	N/A

Reporting for specific materials, systems and methods

Materials & experimental systems

n/a	Involved in the study
☒	☐ Unique biological materials
☒	☐ Antibodies
☒	☐ Eukaryotic cell lines
☒	☐ Palaeontology
☐	☒ Animals and other organisms
☒	☐ Human research participants

Methods

n/a	Involved in the study
☒	☐ ChIP-seq
☒	☐ Flow cytometry
☒	☐ MRI-based neuroimaging

Animals and other organisms

Policy information about studies involving animals; ARRIVE guidelines recommended for reporting animal research

Laboratory animals	22 newborn dairy calves (20 Holstein, 2 Jersey; 5 Male, 17 Female).
Wild animals	The study did not involve wild animals
Field-collected samples	The study did not involve filed-collected samples
